# Primary care receptionists influence migrant access to healthcare by acting as street-level bureaucrats: a scoping review

**DOI:** 10.1186/s12875-026-03201-z

**Published:** 2026-02-28

**Authors:** Georgia Blackwell-Green, Max Cooper, Neil V. Singh

**Affiliations:** 1 Emergency Department, James Paget University Hospital, Great Yarmouth, Norfolk, NR31 6LA UK; 2 Department of Primary Care and Public Health, Brighton and Sussex Medical School, BSMS Teaching Building, University of Sussex, Brighton, East Sussex BN1 9PX UK

## Abstract

**Background:**

Receptionists in primary care play a pivotal, yet underexplored, role in shaping access to healthcare for migrants. This scoping review applies Lipsky’s theory of street-level bureaucracy to examine if and how frontline administrative staff influence migrant access to primary care and thence to further health services.

**Methods:**

A systematic search was done of seven literature databases, the archives of seven journals, and five websites, following guidance by the Joanna Briggs Institute for scoping reviews and the Preferred Reporting Items for Systematic Reviews and Meta-Analyses for Scoping Reviews. We synthesised 44 sources – including peer-reviewed studies, grey literature, and newspaper articles – focusing on migrant interactions with UK general practice receptionists.

**Results:**

Thematic analysis revealed three dominant themes: receptionists are under-prepared to manage linguistic minorities; receptionists feel like protectors of the system; and receptionists’ choices reflect a wider institutional ethos. Findings suggest that receptionists frequently exercise discretionary power to either obstruct, or more rarely facilitate, migrant access to care. Despite official guidance mandating universal access to primary care, migrants are often informally denied registration when they are eligible for care. Receptionists’ behaviours were deeply influenced by implicit biases, the absence of formal translation resources, and prioritising practice priorities over the clinical needs of patients. However, individual actions seem to be shaped by broader discourse and structural constraints.

**Conclusion:**

This review highlights how receptionists exercise personal discretion through which migrant health inequalities are reproduced at the point of access. We make concrete recommendations for changes to medical training and practice, based on our results.

**Supplementary Information:**

The online version contains supplementary material available at 10.1186/s12875-026-03201-z.

## Introduction

The United Kingdom’s National Health Service (NHS) was founded on the principles of universal access and no fees at the point of use. However, in the last two decades there has been a movement towards increasingly restrictive access to healthcare for people not considered ordinarily resident [1–6]. This is ostensibly to be fair to the British taxpayer, by not using finite resources to treat people who have not contributed via taxation [5, 7]. However, the evidence reveals that undocumented migrants place a negligible financial burden on the NHS, and indeed the administrative cost of schemes that go after those who might have wrongly used the NHS exceeds the money it recoups [8, 9]. 

In 2012 then Home Secretary Theresa May announced a series of what she termed ‘hostile environment’ policies [10, 11]. Despite the insistence from the Department of Health that they were not asking health workers to act as border guards, the Immigration Act of 2014 outlined increasingly restrictive entitlements that were mostly based on immigration status, stating that it is everyone’s responsibility to ensure that NHS funds are protected and used appropriately [5, 12–17]. 

Lipsky’s theory of street-level bureaucracy (hereafter SLB) describes the powerful position of front−line public sector workers to reshape public policy on the ground [18]. Public sector workplaces share overarching challenges—in particular limited resources to meet demand and conflicting organizational expectations—resulting in adaptive behaviours that often compromise the human element of the interaction [6, 11, 18–20]. Public sector workers develop their own methods of coping with excessive workload and within this must exercise personal discretion. In order to portray the interactions to the public as uniform and fair, this personal discretion is often disguised as application of protocol. Lipsky defines street-level bureaucrats as:

“Public service workers [who] interact with citizens in the course of the job and have discretion in exercising authority; in addition, they cannot do the job according to ideal conceptions of the practice because of the limitations of the work structure.” ([21], pg xvii)

**Fig. 1:**
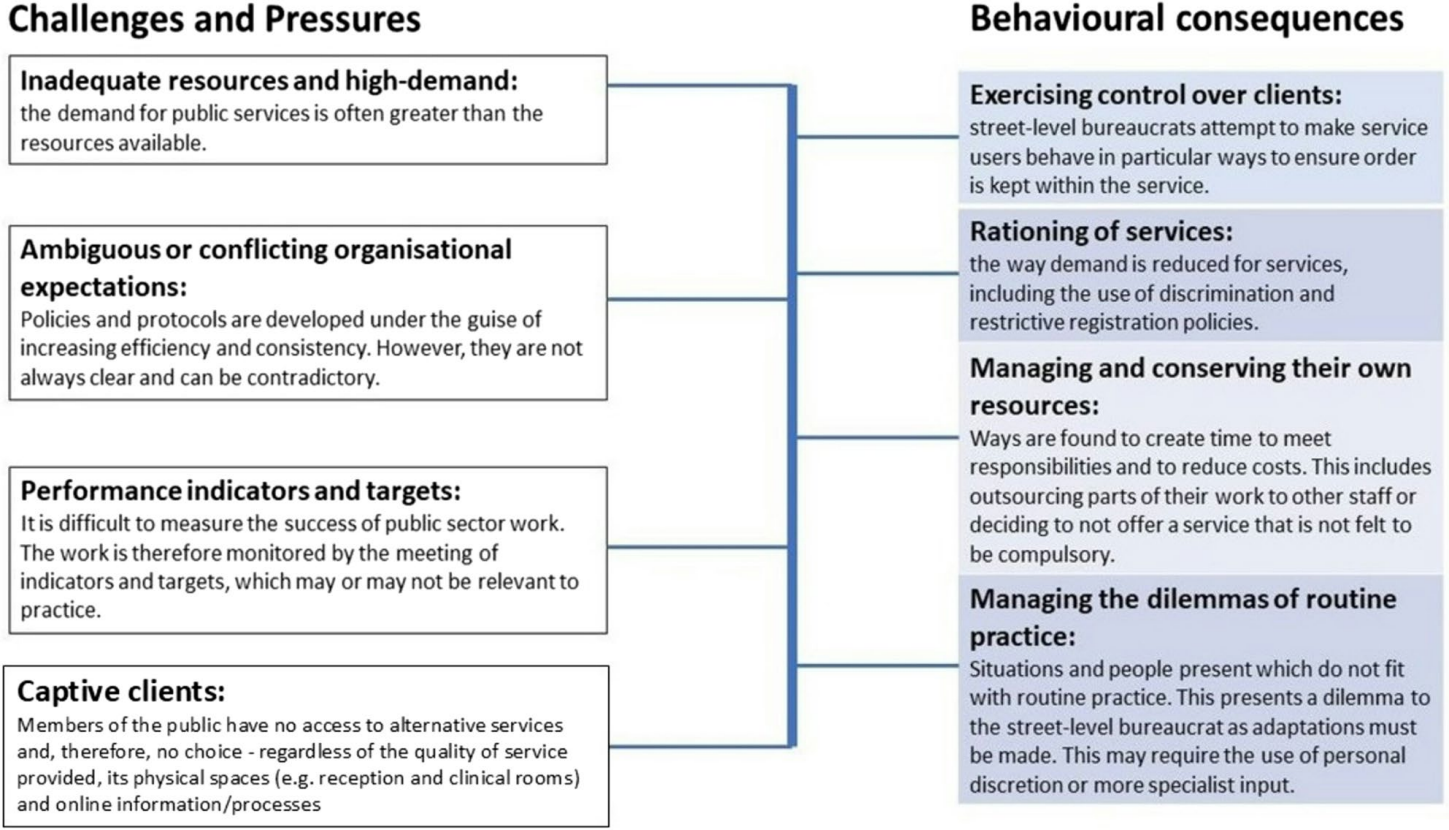
A summary of street-level bureaucracy theory, using titles from Lipsky’s book and Berlan’s SLB summary. Adapted from Lipsky and Berlan et al. [18, 19, 21]

Lipsky regards front−line workers as street−level bureaucrats, and their interactions with the public are crucial in determining how public policy is ultimately delivered [18, 19, 22]. SLB theory is outlined in Fig. 1.

In Lipsky’s original 1980 work, professionals, such as nurses and doctors, were street-level bureaucrats [23]. There has been a body of research viewing General Practitioners (GPs, elsewhere known as Family Doctors or Primary Care Physicians) through SLB theory, both in their patient-facing roles and as service commissioners [24, 25]. GPs find ways to adapt guidelines and apply personal discretion to reduce workload, for example by referring complex patients to secondary care early [24, 25]. However, we believe it is administrative staff, first and foremost receptionists, who occupy a role most closely aligned to Lipsky’s theory.

Receptionists are an under−researched group in healthcare, and their interactions specifically in relation to migrants have been explored even less [26–30]. In General Practice (GP), the receptionist’s role includes: registering (and de-registering) patients; making appointments; informal triage; dealing with questions and complaints; and other administrative tasks [31]. To access a GP, almost all UK patients typically must interact with a receptionist, although in recent years novel methods of triaging care (including digital forms to enter symptoms, and total triage) have been piloted [32]. 

There has been limited research applying SLB theory to reception work [26, 32]. Early research in 1975 by David Hughes highlighted the significant influence receptionists can have in an English Emergency Department [33]. Hughes’ paper explored how healthcare receptionists exercise discretion in the classification of patients, with real impact in terms of the priority in which patients were seen by doctors. This was despite the existence of extensive protocols [33]. 

A 2023 study analysed semi-structured interviews with GP receptionists and placed them within SLB theory [26]. It highlighted the social complexity inherent within reception work. On the one hand aspects of their work were protocol driven, but on the other they could also break protocol for patients they viewed as vulnerable [26, 27]. The paper also emphasised the unique environment in which receptionists work, separate to professional healthcare staff, but in close contact with patients. This can result in frequent, unfiltered exposure to the struggle between supply and demand [26, 33, 34]. This relatively autonomous space means there is potential for receptionists to have informal power, and to develop unique relationships with the patients. Within a medical hierarchy, however, there are marked power imbalances between clinicians (especially doctors) and receptionists [25, 28]. Whether this hierarchy protects patients from under-trained staff having too much power, or rather hamstrings well-meaning front-line workers—limiting their ability to make fair, sensible and kind decisions—is unclear.

Receptionists’ potential to influence access to GP services on the basis of perceived racial and/or migration status has, to our knowledge, never been explored. However, multiple reports have emerged of racialised patients being denied NHS care that they are entitled to [20, 35–39]. A charity-funded health clinic for vulnerable migrants in London found that GP receptionists have incorrectly refused registration for their service users and displayed what they described as worrying ‘gatekeeping behaviour’, controlling access to information and necessary care [36–39]. There have also been similar reports for people that are of No Fixed Address and for Gypsy, Roma and Traveller communities [40, 41]. It is unclear from these existing reports whether such gatekeeping behaviour arises from individual xenophobia, structural racism, or both.

Currently everyone, regardless of immigration status, is eligible to register with a GP and receive primary care, free-of-charge in the UK [42, 43]. It thus felt particularly important to consider how immigration policies and biases (both implicit and explicit) can be enacted in everyday encounters and thus impact the experience of migrants attempting to access primary care services [44]. Accordingly, we aimed to explore the experience of migrants attempting to access primary care in the United Kingdom (UK), through the lens of SLB theory, by conducting a scoping review of available evidence.

## Methods

We chose to conduct a scoping review with a thematic synthesis. This review was performed according to the methodology proposed by the Joanna Briggs Institute for scoping reviews and the Preferred Reporting Items for Systematic Reviews and Meta-Analyses for Scoping Reviews (PRISMA-ScR) [45–47]. 

### Definitions

There is no universally accepted definition for “migrant”, with definitions varying by: length of stay in a country; residency status or possession of relevant documentation; and the reason for migration [48–50]. We adopted the broad definition of migrants, as anyone who has moved to the UK from any other country. The intention is not to portray migrants as a homogenous group, but to acknowledge that immigration status is dynamic and race can interact with other axes of intersectional oppression. By “receptionist”, we refer to the frontline administrative staff that citizens must speak or write to in order to gain further access to the healthcare system.

### Eligibility criteria

Inclusion and exclusion criteria are outlined in Table 1.

**Table 1:** Inclusion and exclusion criteria

**Inclusion Criteria**
• Refers to migrants • Refer to reception and administrative staff directly, or to a recognised role of theirs in the practice e.g. registration or appointment-making • Refer to healthcare access in any way • Comprise qualitative or textual data, including mixed-method studies • Published before 2020 • About, or applicable to, a primary care setting • Relating to the UK
**Exclusion Criteria**
• No clear data from the UK in multi−country analyses (i.e. data from the UK not clearly disaggregated) • Articles solely relating to Northern Ireland • Articles referring to internal migrants (Gypsy, Roma, and other travelling communities) • Articles referring to BAME (Black, Asian, and Minority Ethnic) patients if nationality was British • Solely quantitative studies • Not written in English • Full text not available

We focused our search on literature from the UK, to ensure our analysis would be meaningful and coherent, since different settings would introduce other contextual variables that might account for good or bad access to healthcare.

The Covid-19 pandemic triggered several major changes in how medicine is accessed and practiced. For instance, digital triage and video consultations became commonplace, altering the patient-receptionist relationship. It is for this reason that we decided to cut off our search before 2020, to focus on studies that focus on in person or telephone encounters with reception staff.

The inclusion of grey literature and non−academic literature increased the breadth of the evidence available to be analysed. Including only scholarly articles would have restricted our results, since much of the discourse regarding receptionists in primary care occurs in non-scholarly sites.

### Search strategy

The search terms were divided based on Lipsky’s SLB theory. The *key population* being researched were receptionists and the *secondary population* were migrants. The *contexts* were the UK and Primary Care and the *concept* was access to Primary Care. Northern Ireland was excluded because its health service infrastructure was sufficiently different to introduce additional variables regarding entitlements to care.

The following databases were included in the final selection: MEDLINE, PubMed (PM), Web of Knowledge (WK), Scopus, Social Work Online (SW), Google Scholar (GS), Global Health (GH), and Newspaper (NW, the newspaper database). The journals included in the search were the British Journal of General Practice (BJGP), the British Journal of Community Nursing (BJCN), Sociology of Health and Illness, BMC Family Practice, Contemporary Sociology, BMC Health Services Research, and the Journal of Ethnic and Migration Studies. Websites included the official government website (gov.uk), Doctors of the World, MedAct, BME Health Forum, and the Equality and Human Rights Commission (EQHRC). The full search strategy is included in Appendix. 1.

### Study selection

All studies identified in the search were collected and integrated into Mendeley, duplicates were removed. The lead author (GBG) screened the articles, using the previously defined inclusion criteria. After the assessment of all titles and abstracts, the studies that fulfilled the inclusion criteria were read in full. The full results of the search and the reasons for the exclusion of studies after reading the full text were recorded.

### Data extraction

Data was extracted from selected studies by the lead author using a data extraction tool developed by the reviewers’ team. The data extracted included specific details about the publication, the concept, context, study methods, and relevant findings. There was no need to contact the authors of the included articles for further information or data clarification.

### Data analysis and presentation

Relevant data was extracted in tabular format, and a thematic analysis accompanies the results, describing how the results relate to the stated aims of this review. Thematic analysis was performed by hand by the lead author, guided by both Burnard’s method for thematic analysis for primary qualitative data and Thomas and Harden’s proposed method for undertaking thematic synthesis of qualitative data for systematic reviews [51, 52]. SLB theory (Fig. 1) was then applied to the themes to provide an understanding of how receptionists behave as street−level bureaucrats on the front line in their interactions with migrants.

## Results

**Fig. 2:**
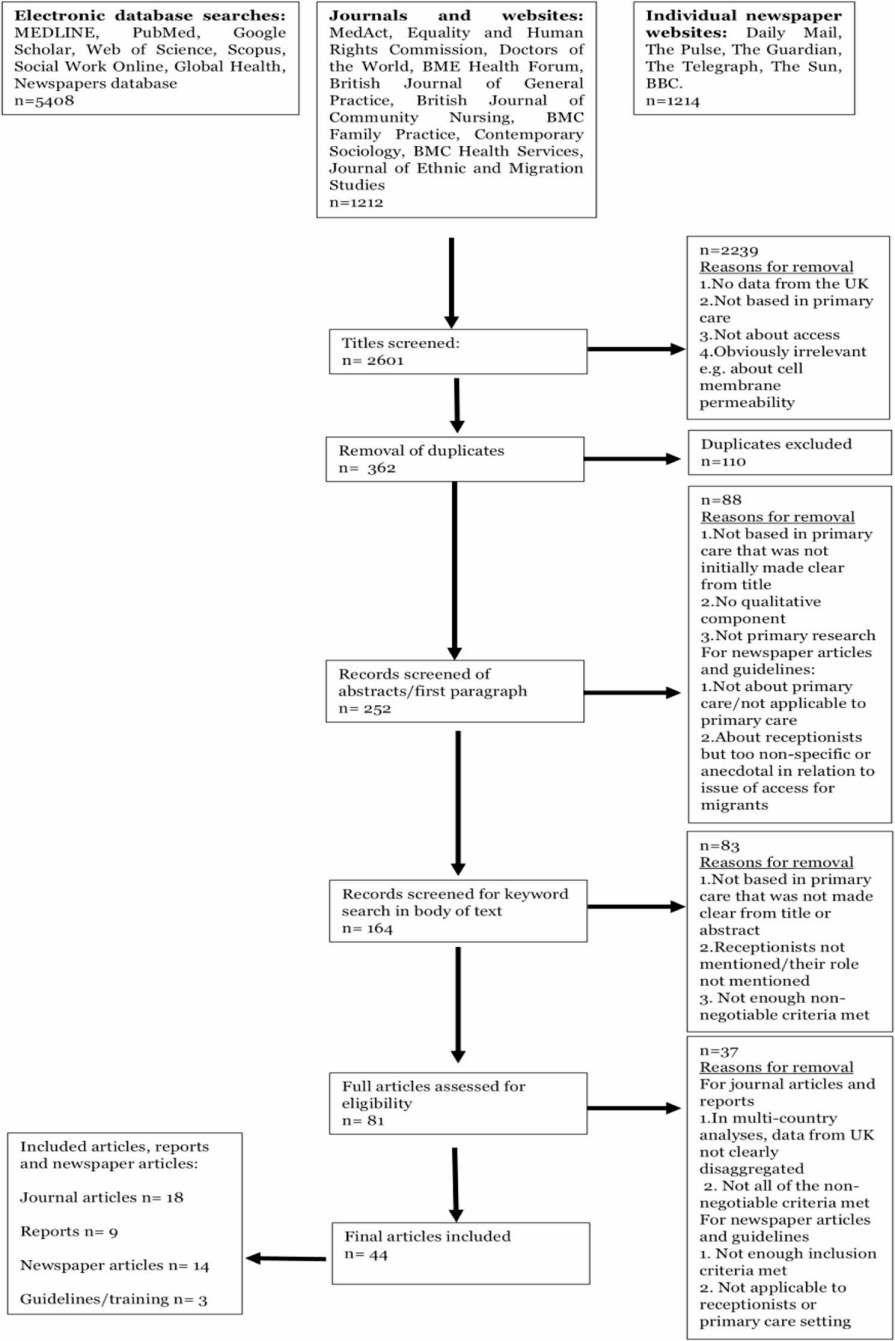
PRISMA flowchart for the search strategy and process of study selection. Adapted from the Joanna Briggs Institute Model for Evidence-Based Healthcare [46]

A total of 44 articles were included in the final analysis. 41% were journal articles (*n* = 18), 32% (*n* = 14) were newspaper articles, 13.6% (*n* = 6) were reports from advocacy organisations, 6.8% (*n* = 3) were reports from the Department of Health, 4.5% (*n* = 2) were guidelines from advocacy organisations and 2.2% (*n* = 1) were guidelines from the Department of Health. Of all the documents analysed, only 15.6% involved interviewing receptionists directly (4 journal articles, 2 reports and 1 newspaper article). 38% of all included documents focused on the migrant’s experience of accessing primary care (10 journal articles, 7 reports and 1 newspaper article). A summary of the search strategy and selection process is presented in the PRISMA flowchart in Fig. 2.

The date range of the articles included is from 1985 to 2019, with 60% published from 2013 onwards, since the introduction of more restrictive “hostile environment” health policy. The migrant populations mentioned or interviewed in the articles (with the number of corresponding studies in brackets) were: asylum seekers [9], refugees [5], refused asylum seekers [3], undocumented migrants [3], trafficked individuals [2], recently−arrived migrants [1], European migrant families [1], Black and Minority Ethnic migrants [1], and unspecified migrant groups [11]. The included articles are summarised in Table 2.

**Table 2:** Selected characteristics of the included articles

First author, Year (Reference Number)	Title	Journal/ website (and type of source)	Aims	Study population	Location	Method	Analysis conducted
Abdulkadir et al. 2016 [53]	What do you mean, I have a right to health? Participatory action research on health and human rights	Health and Social Care Action Group (*Report*)	n/a	34 people with experience of homelessness and 49 women with refugee status or asylum seeker (83 total)	Glasgow	Focus groups discussions based around the PANEL human rights principles (Participation, Accountability, Non−discrimination, Empowerment and Legality)	Thematic analysis and identified themes categorised as positive, negative or neutral according to the participant perspectives.
Arber & Sawyer 1985 [29]	The role of the receptionist in general practice: a dragon behind the desk?	Social Science and Medicine (*Journal article*)	To analyse the influence of the organisation of primary care on patient’s experience of and attitudes towards that care	1000 adults filled in a survey and a smaller sample [26] from the electoral register selected to interview	South East England – specificall y SW Thames regional health authority, Surrey and Merton − Sutton and Wandswo rth Area	Patient survey and interview	Comparison between care provided in different ‘types of practice’. Exact method of statistical analysis and qualitative analysis not specified.
Bates 2016 [54]	The GP surgery that treats patients no one else can cope with	BBC (*Newspaper article*)	n/a	Asylum seekers, GPs, other patients that struggle to access mainstream services	Huddersfield	Article describing a practice that takes on patients who have been de− registered or rejected by mainstream services.	n/a
Bhatia & Wallace 2007 [55]	Experiences of refugees and asylum seekers in general practice: a qualitative study	BMC Family Practice (*Journal article*)	To explore whether the available guidelines and training initiatives make sense to migrants and other key stakeholders and whether they could collectively choose guidelines and training initiatives and engage in their implementation in primary care settings.	Asylum seekers, refugees, refused asylum seekers. 11 participants.	Barnet, London. Walk − in clinic.	Semi− structured interviews	Framework analysis
BME Health Forum 2008 [56]	Primary concern: access to GP practices for black and minority ethnic communities in Kensington, Chelsea and Westminster	BME Health Forum (*Report*)	To understand the systems involved in registering with PC practices and making appointments, whilst considering how the specific characteristics of the BME communities may impact upon their use of health services. To assess the confidence that various stakeholders have in the interpreting services; and to understand how these variations impact upon the use of health services. To consider the quality and nature of the relationship between GPs and patients; to understand any relationship issues that may impact upon the provision and uptake of health services	6 different groups of stakeholders. 1.Patients from a BME/migrant background with differing English language levels, 2.General Practitioners (GPs) 3.Practice managers 4.Chairs of PBC Clusters 5.Clinical/Professi onal Executive Committee Chairs (for each PCT) 6.BME Health Forum Steering Group and patient representatives,	Two boroughs in London – Kensingto n and Chelsea	Questionnair es and focus groups	Actual analytical method not made clear in report. States ‘patient questionnaire was analysed as a whole sample, but also separately for each of the groups… The analysis is formed into 3 sections registering and making appointments with a GP, accessing and the quality of Interpreter services, the nature and quality of the interpersonal relationship of the GP and the patient.’
Borland 2017 [4]	Health tourism crackdown	Daily Mail (*Newspaper article*)	n/a	n/a	England	Report about a new policy for PC to collect EHIC information	n/a
Carlowe 2001 [57]	The doctor won’t see you now	The Guardian (*Newspaper article*)	n/a	Contains quotes from asylum seekers, asylum advocate, British Medical Association representative	England	n/a	n/a
Chan Aung et al. 2010 [58]	Access to and utilisation of GP services among Burmese migrants in London: a cross− sectional descriptive study	BMC Health Services Research (*Journal article*)	To assess knowledge of Burmese migrants on health services in Greater London, the current level of access to and utilisation of PC services, barriers or obstacles encountered during PC registration and when consulting General Practitioners, and socio−demographic disparities in access to health care within Burmese migrants.	Burmese migrants from 3 different areas of London. All of different immigration statuses. 137 participants.	London	Self− administered questionnair es (137 returned) and in−depth interviews (5 selected post−survey)	SPSS used to analyse survey data. Descriptive analysis, multivariate analysis, Pearson’s correlation test and Chi square test conducted on survey data. Content analysis used on interviews.
Creative Research 2013 [1]	Qualitative assessment of visitor and migrant use of the NHS in England	Department for Health (*Report*)	To provide DH with a better understanding of how key NHS stakeholders perceive the issue of migrant and overseas visitor use of the NHS in England, by engaging with a wide range of clinicians in primary and secondary care as well as managers and administration staff across England	Qualitative assessment took place across primary care and secondary care. Trusts grouped based on how well they had implemented the cost recovery programme and a sample from each group tried to be obtained. Overseas visitors managers included even though they are secondary care as they spoke about interacting with primary care. 14 primary care practices participated which included 62 members of staff (practice managers, deputy practice managers, practice nurses, reception managers, reception staff, administration and secretarial staff, and a family health worker. 3 clinical commissioning groups also agreed to take part.	England	Interviews, workshops, discussion groups	Thematic analysis
Daniels 2010 [59]	GP receptionists are invaluable	The Guardian (*Newspaper article*)	n/a	Opinion piece by GP	United Kingdom (does not specify location)	Opinion piece based on experience working in a GP surgery	n/a
Department of Health 2015 [60]	Cost recovery training programme e−learning	Health Education England (E−Learning presentation aimed at NHS administrative staff)	n/a	n/a	n/a	n/a	n/a
Doctors of the World 2015 [39]	Registration refused: a study on access to GP registration in England 2015	Doctors of the World (*Report*)	To explore the barriers to registration for service users with GPs To establish the prevalence of poor practice among PC practices in the registration of DW patients, the reasons for registration refusal and consistency within and between practices with regard to registration refusal.	Migrants accessing DoW clinic, GP practice staff	London	Looks at case notes collected by volunteers when trying to register service users with GP practices	Basic statistics, use of case studies and quotes from reception staff to contextualise findings.
Doctors of the World 2016 [37]	Registration refused: a study on access to GP registration in England Update 2016	Doctors of the World (*Report*)	To explore the barriers to registration for service users with GPs To establish the prevalence of poor practice among PC practices in the registration of DW patients, the reasons for registration refusal and consistency within and between practices with regard to registration refusal.	Migrants accessing DW clinic, GP practice staff	London	Looks at case notes collected by volunteers when trying to register service users with GP practices	Basic statistics, use of case studies and quotes from reception staff to contextualise findings.
Doctors of the World 2017 [36]	Registration refused: a study on access to GP registration in England Update 2017	Doctors of the World (*Report*)	To explore the barriers to registration for service users with GPs to establish the prevalence of poor practice among PC practices in the registration of DW patients, the reasons for registration refusal and consistency within and between practices with regard to registration refusal.	Migrants accessing DW clinic, GP practices	London	Looks at case notes collected by volunteers when trying to register service users with GP practices	Basic statistics, use of case studies and quotes from reception staff to contextualise findings.
Doctors of the World 2018 [38]	Safe Surgeries Toolkit	Doctors of the World (*Toolkit/guida nce for practice staff to facilitate access to primary care*)	n/a	n/a	n/a	n/a	n/a
Doctors of the World 2014 [20]	Guidelines for patient registration (receptionist)	Doctors of the World (*Poster directed at receptionists*)	n/a	n/a	n/a	n/a	n/a
Dunne 2002 [61]	Analysis: GPs and asylum seekers	BBC (*Newspaper article*)	n/a	Asylum seekers, GPs. Contains quotes from GPs working at the Medical Foundation for the Care of Victims of Torture, the Sanctuary	Birmingham, Hackney	News report detailing how a GP closed their doors to asylum seekers and refugees	n/a
Fang et al. 2015 [62]	Experiencing ‘pathologised presence and normalised absence’; understanding health related experiences and access to health care among Iraqi and Somali asylum seekers, refugees and persons without legal status	BMC Public Health (*Journal article*)	To explore health and health care experiences of Somali and Iraqi asylum seekers, refugees and persons without legal status To use minoritisation and pathologisation processes as an analytical lens to understand the multiple layers of oppression that contribute to health inequalities	Iraqi and Somali asylum seekers, refugees and persons without legal status. 35 participants.	Manchest er, United Kingdom	Focus groups and in−depth interviews	Phoenix’s theory of marginalisation and othering used as analytical framework
Gregory 2017 [7]	Why isn’t the NHS making patients pay?	The Daily Mirror (*Newspaper article*)	n/a	n/a	England	Report on GP policy to identify chargeable patients	n/a
Grit et al. 2012 [63]	Access to health care for undocumented migrants: a comparative policy analysis of England the Netherlands	Journal of Health Politics, Policy and Law (*Journal article*)	To analyse the contextual, institutional, or actor related factors that have influenced health care policy development on undocumented migrants in England and Netherlands	Health care professionals, policy makers, NGOs, professional medical associations	England and Netherlands	In−depth interviews and policy analysis	Policy analysis – interviews used as case studies.
Hammond et al. 2013 [27]	Slaying the dragon myth: an ethnographic study of receptionists in UK general practice	British Journal of General Practice (*Journal article*)	To explore the complexity of the role of general practice receptionists by considering the wider context in which they work	45 receptionists across 7 practices	Northwes t England	Ethnography (200 h of observation and conversation s with staff)	MAXQDA analysis software used, codes given to ‘meaningful incidents’ and related incidents grouped together.
Hargreaves et al. 2007 [9]	Charging systems for migrants in primary care: the experiences of family doctors in a high−migrant area of London	Journal of Travel Medicine (*Journal article*)	To look at the impact of Overseas Visitor use on PC practices in a high migrant area of London, and to see how changes in the charging regulations may impact them − how are they current set up to identify overseas visitors	GPs	London borough of Newham,	Surveys and semi− structured interviews	SPSS, chi− squared test and thematic analysis
Ingram 2009 [64]	The health needs of the Somali community in Bristol	Community Practitioner (*Journal article*)	To identify the health needs of the Somali community in Bristol by conducting 10 semi structured interviews with community representatives and HCPs, and two focus groups with Somali residents	Somali residents of Bristol and health care professionals, key Somali community representatives	Bristol	Semi− structures interviews and focus groups	Qualitative rapid appraisal
Ipsos Mori Social Research Institute 2017 [12]	Overseas visitor and migrant NHS cost recovery programme: formative evaluation − final report	Department for Health (*Report*)	To determine how far the Cost Recovery Programme has led to the desired changes in culture and behaviour amongst frontline clinical and administrative staff (and other relevant stakeholders) with regard to practices for identifying and recovering costs from overseas visitors and migrants using NHS services; To learn lessons about what works (and doesn’t work) in improving cost recovery, including through analysis of the Emergency Care EHIC Pilot; To help refine the Cost Recovery Programme through continuous feedback and inform decisions before proceeding with each stage of the Cost Recovery Programme	NHS staff in primary care and secondary care across all roles including overseas visitors managers, admin staff, nurses, doctors, practice managers.	13 Trusts across England	In−depth interviews, case studies, quantitative indicators of behavioural and cultural change and telephone surveys conducted each year across 3 years of implementat ion.	Basic statistics and summarising of key points from interviews and case studies.
Kang et al. 2019 [65]	Access to primary health care for asylum seekers and refugees: a qualitative study of service user experiences in the UK	British Journal of General Practice (*Journal article*)	To examine asylum seeker and refugee experiences accessing primary health care in the UK in 2018	Asylum seekers, refugees and refused asylum seekers. 6 refugees, 8 asylum seekers and 4 refused asylum seekers. 18 participants altogether.		Semi− structured interviews	Penchancky and Thomas framework of access used to analyse thematically.
Lind 2013 [66]	GPs to police access to NHS care under plans for new registration system for migrants	The Pulse (*Newspaper article*)	N/A	Contains quotes from Jeremy Hunt (then Health Secretary)	United Kingdom	Article describing Hunt’s proposed changes to registration systems in primary care in order to increase charge recovery from ‘ineligible’ patients	n/a
Lindenmeyer 2016 [28]	Experiences of primary care professionals providing healthcare to recently arrived migrants: a qualitative study	British Medical Journal (*Journal article*)	To explore the experiences of primary care professionals providing care to recent migrants in a superdiverse city and to elicit barriers and facilitators to meeting migrants’ care needs.	6 GPs, 5 practice nurses, and 6 administrative members of staff across 10 practices	Birmingh am	Semi− structured interviews	N−Vivo software used to conduct thematic analysis.
Lionis et al. 2015 [67]	Engaging migrants and other stakeholders to improve communication in cross−cultural consultation in primary care: a theoretically informed participatory study	British Medical Journal (*Journal article*)	To explore whether the available guidelines and training initiatives make sense to migrants and other key stakeholders and whether they could collectively choose guidelines and training initiatives and engage in their implementation in primary care settings	Migrants, general practitioners, nurses, administrative staff, interpreters and health service planners. 78 participants.	Participan ts from Austria [15] England [9] Ireland [11] Greece [16] Netherlan ds [27]	Focus groups using participatory learning action	Thematic analysis
Lockley 2019 [68]	The NHS is ignoring guidelines and refusing to treat thousands of people	The Canary (*Newspaper article*)	N/A	n/a	England	Mystery shopper exercise – people rang around practices to see if they could register without documentati on or proof of address	n/a
Moss 2013 [69]	Pills, bills and bellyaches: a peek behind the scenes at a GP surgery	The Guardian (*Newspaper article*)	N/A	Contains quotes from reception staff, GPs, the deputy practice manager	England	Observation and interviews – exact method not described but the journalist spent several days sitting in on the running of a GP practice with different staff members,	n/a
Nellums et al. 2018 [35]	The lived experiences of access to healthcare for people seeking and refused asylum	Equality and Human Rights Commission (*Report*)	To examine the barriers and enablers experienced by people seeking and refused asylum when they try to use health services in Britain To contribute crucial information on the reality of accessing healthcare for these specific groups, and help fill recognised evidence gaps	People seeking and refused asylum and service providers. 21 participants were asylum seekers, 9 participants had been refused asylum. Representatives from 31 different organisations were involved in the project.	London, Swansea, Nottingha m, Glasgow	Focus groups discussions, one − to−one interviews	Thematic analysis
Offredy 2002 [70]	Access to primary care: decision making by GP receptionists	British Journal of Community Nursing (*Journal article*)	To examine decision making by receptionists when allocating patients to consult either the General Practitioner or the Nurse Practitioner. It also addressed receptionists’ decision making within the context of current government policies.	15 GP receptionists across 15 GPs	South East England	Interviews and observation of a shift	NUD*IST qualitative data analysis software Programme used to analyse the data. Systematic coding using the interview questions as a framework to categorise codes.
O’Donnell et al. 2006 [71]	‘They think we’re OK and we know we’re not.’ A qualitative study of asylum seekers’ access, knowledge and views to health care in the UK	BMC Health Services Research (*Journal article*)	To better understand the barriers and health needs of the asylum seeker population in Glasgow	Asylum seekers	Glasgow	Focus groups and interviews	Framework analysis
Oram et al. 2015 [72]	Provider Responses to Treatment and Care of Trafficked People	Department of Health (*Report*)	To understand what types of health services trafficked people had used, how they had accessed them and their experiences of using these services.	160 trafficked people participated as part of the PROTECT research programme. 140 participated in a qualitative interview at the end of the survey.	England	Interviews following survey	N−Vivo software used to analyse data. Framework analysis.
Poduval et al. 2015 [3]	Experiences among undocumented migrants accessing primary care in the United Kingdom: a qualitative study	International Journal of Health Services (*Journal article*)	To explore the experiences of undocumented migrants trying to access primary care in the UK, their perspectives on proposed restrictions and to make suggestions for policy makers	Undocumented migrants and volunteer staff at charity clinic. 16 participants were undocumented migrants and 4 were charity staff.	Charity clinic in London	Semi− structured interviews	Framework analysis
Rafighi et al. 2016 [73]	National health service principles as experienced by vulnerable London migrants in “Austerity Britain”: a qualitative study of rights, entitlements and civil−society advocacy	International Journal of Health Policy and Management (*Journal article*)	To explore policy reform challenges and implications, using excerpts from the perspectives of non − EEA migrants and health advocates in London	Non − EEA migrants and civil society advocates. 16 participants were non − EEA migrants and 6 civil society advocates.	London	In−depth interviews	Thematic analysis
Robinson 2016 [74]	Do NHS receptionists put you off seeing your doctor?	The Guardian (*Newspaper article*)	N/A	Receptionists, patients at GP practices	United Kingdom (does not specify location)	Article based on a survey by Cancer Research UK about patient satisfaction with GP receptionists	
Slawson 2016 [75]	Hackathon designs app to help migrants navigate the NHS	The Guardian (*Newspaper article)*	n/a	n/a	United Kingdom (does not specify location)	News report about a phone app competition. The winners developed a downloadabl e letter to be shown to receptionist when trying to register at a GP surgery.	n/a
Tesfaye & Day 2015 [76]	Health visitors’ perceptions of barriers to health and wellbeing in European migrant families	Community Practitioner (*Journal article*)	To explore health visitor perceptions of barriers to health and wellbeing faced by European migrant families and common challenges experienced in practice	Health visiting professionals. working with European migrant families. 8 participants.	Merseyside	Semi− structured interviews	Thematic analysis using theoretical constructivist approach
Unknown 2011a [77]	GPs treating asylum seekers unfairly targeted by PCOs	The Pulse (*Newspaper article*)	n/a	n/a	UK	Describes how some practices who have registered undocument ed migrants are being investigated by the primary care trusts.	n/a
Unknown 2011b [78]	GPs forced to register illegal immigrants after threat of legal action	The Pulse (*Newspaper article*)	n/a	n/a	UK	Describes the case of a practice who deregistered a couple once they discovered they were undocument ed migrants. Contains interview excerpts from Practice Managers at the practice.	n/a
Unknown 2016 [6]	NW.02.52 Q&A	Daily Mail (*Newspaper article*)	n/a	n/a	UK	A short piece answering questions from the public about who should check patient’s right to access NHS care.	n/a
Warfa et al. 2006 [79]	Post−migration geographical mobility, mental health, and health service utilisation among Somali refugees in the UK: a qualitative study	Health and Place (*Journal article*)	To understand why residential mobility in the ‘host country’ may be associated with poor mental health for refugee populations and Somali migrants’ perceptions on the relationship between residential mobility, poor health and health service use	Somali professionals and ‘lay Somalis’. 13 Somali professionals and 21 ‘lay Somalis’ participated. 34 altogether.	East and South London	Discussion groups	Framework analysis
Westwood et al. 2016 [2]	Access to and experiences of healthcare services by trafficked people: findings from a mixed methods study in England	British Journal of General Practice (*Journal article*)	To explore trafficked people’s access to and use of health care during and after trafficking.	People with experience of being trafficked, who were no longer being exploited. 136 participants.	England	Structured survey questions and interviews	N−Vivo software used to conduct thematic analysis.

Thematic synthesis identified three core themes: receptionists are under-prepared to manage linguistic minorities; receptionists feel like protectors of the system; and receptionists’ choices reflect a wider institutional ethos.

### Receptionists are under-prepared to manage linguistic minorities

At reception a language barrier could create significant challenges to completing the most routine of tasks. It affected registration, appointment−making, information dissemination and created administrative barriers [1, 58, 64, 67, 75, 76, 79]. In practices where interpreter services were available for healthcare professionals, they were not available at the reception desk.

*“Is the [language] training not open to frontline staff also*,* as they are the ones who have first contact with service users?” [Receptionist English GP practice]* [67]

The language barrier could result in routine interactions becoming time consuming:

*“Same thing for reception*,* it depends who comes in on the morning. You could spend 20 minutes*,* we have spent 20 minutes with one patient maybe just trying to book an appointment because they just don’t understand*,* we don’t understand*,* they don’t understand*,*” [Receptionist]* [1]

There were examples of receptionists using resources available to them, such as Google Translate. Some receptionists highlighted how using Google Translate to say just a few greeting words in the language of the patient helped to put people at ease [28]. 

However, the most common scenario described was the reliance of migrants on social contacts, who could assist with translation:

*“First time my English was bad*,* they didn’t listen us because they said they no understand me and no translator. Have to find friend to bring me and explain what’s problem.”* [55]

Some practices had a telephone-only system in place, which was more challenging to navigate:

*“We didn’t have telephone. But.reception say you don’t come*,* you have to call. We can’t speak on phone. If you see on the face it’s easier” [Asylum seeker discussing communication difficulties when booking appointments over the phone]* [55]

Receptionists could also be frustrated with unsuitable protocol:

*…anyone collecting prescriptions on behalf of someone else had to sign their own name*,* as well as writing the name of the patient. However*,* some couldn’t write English and most receptionists couldn’t read Urdu… Receptionists reported being tempted to break the rules by writing the patient’s name themselves.* [27]

A cycle was created whereby receptionists were unable to offer language support or disengaged, leading to a reliance on external supports to navigate the service and a lack of accountability to incite change [35, 55, 58]. 

### Receptionists feel like protectors of the system

The protective role of receptionists took various forms, such as protecting resources for patients considered vulnerable, or protecting the practice from repercussions both financial and punitive [1, 7, 12, 14, 27, 54, 59, 61, 70].

*Receptionists considered protecting the system*,* and by extension the vulnerable patients*,* from those attempting to take advantage of it to be one of their central responsibilities.* [27]

There were examples of receptionists being flexible with protocol, for patients they considered vulnerable. In cases where refugees or asylum seekers were considered vulnerable, receptionists would reassure of their entitlement to register [27, 54, 59]. However, migrants, including asylum seekers and refugees, could also be viewed as a burden to the practice, from which the practice needed to be protected [1, 17, 61, 70, 78].

*On another occasion when trying to register so she [female asylum seeker] could book an appointment for an urgent health concern*,* the receptionist*,* after refusing to register her said*,* “Why do you worry? The Home Office gave you a house*,* and money for eating.”* [35]

There were also concerns that registering asylum seekers could lead to government-funded Quality Outcome Framework targets being missed as they were more likely to be living in temporary housing.

*“Because the patient’s still registered*,* we miss all the targets because they’re not at that address or they’ve moved on because they’re transient patients.”* (*Practice partner*,* practice managers & reception manager*,* PC20a).* [1]

Registering migrants as temporary patients was viewed as a compromise by practice staff to accommodate both the priorities of the practice, whilst still placating the individual attempting to register.

The perception that registering migrants damaged the financial viability of the practice could lead to a decision to only support migrants who met the practice requirements, and an insistence that migrants must adapt to the practice [7, 9, 28, 73]:

*“If you want to see a doctor and we have to meet your demands*,* you will see any doctor. They really have now to mould to our service*,* and this is what they are not understanding— they won’t change.” [Admin staff]* [28]

In quotes like this we see evidence of receptionists being unaware of the importance of continuity and trust when socially marginalised groups seek medical care, assuming “standard care” is suitable for migrants too.

### Receptionists’ choices reflect a wider institutional ethos

In some practices there was a shared perspective amongst the receptionists, seeing migrants as burdensome patients; this group mentality emboldened individuals to act in ways they may not have without a critical mass of like-minded colleagues [1, 27, 63, 70, 80]. 

The enforcement of routine administrative policy was frequently used as a reason to decline registration:

*upon presenting to a GP surgery to register*,* she [asylum seeker] was asked for documentation she didn’t have*,* and after explaining she should be able to register*,* a receptionist told her “I’m just reception. You go away.”* [35]

However, even within the same practice, if an individual attended with an advocate, or a friend, their experience could be different:

*It became apparent that only those who were accompanied by a friend*,* relative or refugee agency staff member experienced a trouble−free registration process.* [55]

In other cases, when a receptionist was unsure, the decision was escalated to a senior:

*“[The receptionist] refused registration due to requesting proof of address; said we would provide DOTW [Doctors of the World] letter. Checked with manager and told SU [service user] to bring letter and manager would decide then.”* [39]

A lack of knowledge within a practice on people’s entitlements to register, played a part in individuals being declined registration. However, the change in registration success when attending with an advocate, and examples of an unwillingness to learn of people’s rights, suggest that at least some of these decisions may be reflective of a wider practice ethos [17]. 

The wider practice ethos included all layers of the workforce within a practice, including GPs. Doctors are stated to have a duty of care to patients and have the discretion to treat people, regardless of rights or entitlements [5, 81]. However, registration decisions are almost always made before a doctor becomes involved. This was sometimes posed as outside of the doctors’ control:

*However*,* although doctors may want to treat their patients*,* several interviewees indicated that this could prove difficult because patients often have to get past frontline staff members who may require certain documentation.* [63]

However, doctors could influence routine practice in the reception space, when it suited them to do so. For example, in one practice a GP wanted reception to make note of the patient’s clinical reason for booking an appointment. Some receptionists felt uncomfortable doing this as patients could get angry with them. However, the doctor would publicly reprimand the receptionists if their request was not met [27]. 

This highlighted that it was not outside of the remit of healthcare professionals to enter the reception space to ensure correct practices were taking place [27]. Healthcare professionals may be content to let receptionists make access decisions on the front−line and then frame it as a desire to focus on providing medical care.

*Currently there are significant numbers of GPs who exclude undocumented migrants from registering because they do not wish to provide free treatment.* [63]

GPs could be complicit in allowing discriminatory practices at the front-desk to continue:

*“I usually send the refugees to the [general practice] at the end of the road. I just tell them we’re full up or we’re not taking on any more patients. Dr K knows I do it*,* but I don’t tell him every time I do it.” [Receptionist]* [70]

There was also evidence that receptionists felt they knew the types of patients the doctor wanted to see; ‘creaming off’ patients whom GPs considered readily suitable to receive care. The receptionist portrayed the decision as common−sense, but these decisions were arbitrary, or at least not linked to clinical urgency [21, 41, 70]. 

Routine practice in alignment with guidelines could be used as a reason to decline registration [1, 5, 12, 14, 76, 78, 81–83]. However, there appeared to be careful selection as to which guidelines were adhered to:

*The receptionist…when told about the NHS England guidelines*,* she said: “Well*,* that’s NHS England*,* this is our practice policy.”* [36]

Sometimes, plain untruths were employed to dissuade “problem patients” from registering (patients are legally not required to provide any documentation to register with a GP]:

*They said they cannot register anyone without an ID and that their clinic has its own policy.* [36]

The ‘Safe Surgeries Toolkit’ developed by ‘Doctors of the World’ highlights that an inclusive practice ethos can be created, whilst still complying with legal requirements and entitlements [83]. However, the ‘Cost Recovery Programme’ has a vision for the future, whereby the identification of chargeable patients will start within primary care, and that it should become second nature for receptionists to do this:

*Front−line clinical and administrative staff play a role in cost recovery without being aware that changes are driven by a centrally coordinated programme*,* provided the changes in administrative processes are integrated into routine activity.* [13]

Despite multiple examples of decisions regarding registration communicated as inflexible, there was evidence of practices taking place outside of protocol, where personal discretion is used. The perception that practices can effectively follow their own policy, highlighted that internal decisions within a practice can have significant impact on the realisation of entitlements into access for migrants [28, 37, 61, 77]. There appeared to be little understanding at the government level that receptionists work with discretion and power, as street-level bureaucrats, or that policy implementation in public services inherently involves compromises and work-arounds [16, 21]. 

Receptionists in primary care are confronted by people with urgent health needs and differing social circumstances that policies do not account for [13, 41, 80]. They must develop their own methods of coping with the variation in their work, whilst simultaneously portraying it as an application of the policy [26, 33]. The practice ethos created within the reception space shaped at least the initial part of the experience of entitlements to primary care for migrants [36, 53, 55, 56, 65]. Although the reception space could be relatively autonomous, it was also influenced by the practice as whole. Hence decisions regarding registration, communicated by receptionists, could be reflective of a wider practice ethos.

## Discussion

### Summary

Our aim was to explore the experience of migrants attempting to access primary care in the UK. This review confirms the important role receptionists play in UK general practice. Receptionists are not impartial and passive gatekeepers executing neutral policy. Rather, they are crucial social actors with the potential to aid or impede eligible migrants from attaining registration and medical care [26, 57, 70, 72]. On the one hand, reception work can be portrayed as menial and protocolised; on the other hand, there are multiple examples of receptionists exerting their power to directly impact migrants’ ability to register and access primary care, both positively and negatively, especially when supported by other practice staff. This can be linked to Lipsky’s SLB theory, and to wider literature [28, 35, 54, 59]. 

Our results highlight that GP receptionists are under-prepared to manage linguistic minorities. The shortage of official language support at reception leads to a reliance on informal social supports (which is not recommended as it impedes confidentiality and impartial translation) or advocacy organisations to translate [35–37, 39]. As it is time-consuming for receptionists to complete routine tasks with the limited language resources available to them, receptionists often redirect their own resources to English-speaking patients, or disengage from interactions with linguistic minorities, resulting in the individual not attempting to register at the practice [35]. 

Migrants then share information about which practices were unwelcoming within their own social circles. Ironically, this might suit practices who prefer not to register “problem patients” who may require additional time and resources than English-speaking patients. Hence fewer migrants attempt to register overtime, leading to late presentation of illness in Emergency Departments, and preventable health conditions being managed late, in moments of crisis [35–37, 39, 84, 85]. 

Our review also highlights the highly complex social role played by receptionists, perceiving themselves as protectors of the system. Asylum seekers, refugees, and undocumented migrants are disproportionately affected by both official and unspoken restrictive registration policy [25, 35]. The degree to which this policy is used to specifically target patients that are considered burdensome to the practice is difficult to establish, but it is supported by anecdote and some prior literature [17, 86, 87]. 

It is possible that migrants are being turned away due to the context of high demand for services, with limited resources; receptionists may feel the extra time and resources required and deserved by migrant patients could be better spent on a larger number of mainstream British patients [1, 28, 54, 56, 66, 70, 77, 78]. This is beyond the stated job remit of receptionists, but “the system” (both the practice and the health system as a whole) may have an ulterior motive to ignore such social triage, as it may keep migrant health needs (and so their draw on resources) off radar [17, 26, 32]. Some receptionists, wrongly, demand documents from all members of the public attempting to register. However, there is evidence that demands for documentation, as part of registration, remain in place because it knowingly screens-out patient groups perceived to be “burdensome” or less “deserving.” [17, 35, 54, 56, 64, 88] Other approaches described by migrants to deter registration included the use of rude language and dismissive behaviour, gatekeeping information or lying that a practice was not accepting new patients [17, 57, 65, 70, 72, 75]. 

There are, however, also examples of good, inclusive practice by receptionists. One practice described how instead of migrants having to adapt to the practice, the practice adapted to them [28]. How migrant-friendly practices were, seemed to depend on three key components: accurate knowledge on migrant’s entitlements to register, ideas around migrants’ “deservedness” to NHS resources, and finally whether migrants were considered vulnerable for whom resources needed to be conserved [17, 28, 35, 37, 56, 78, 83]. In practices with a migrant-friendly ethos, there was awareness amongst some receptionists of BMA registration guidelines, and they were able to reassure migrants of their rights to register without proof of ID or address [37, 54]. 

This leads to the final theme. If GP partners and managers are reluctant to register patients classed as time consuming, then this is likely to promote discriminatory behaviours at the front-desk. Hence it is possible for practices to create an environment where reception staff are aware of unwritten rules regarding which types of patients the GP wants to see [26, 70]. In Lipsky’s words:

“there is structural receptivity to prejudicial attitudes. The need for simplification exists, so to speak, prior to the stereotype. The stereotype is nurtured in a context where it functions to divide up the client population.” ([21], pg 115)

Receptionists’ way of managing the ambiguities of routine practice was to develop informal policies of their own. This could lead to “common-sense” decisions which are outside of protocol, but where receptionists felt confident what was expected by the wider practice [26, 33, 70]. 

The labelling of migrants as demanding and difficult relates to Jeffrey’s 1979 work ‘*Normal rubbish: deviant patients in casualty departments*,*’* which describes how staff create negative labels for patients who do not conform to the rules of the Emergency Department [80]. Receptionists must balance the expectation of the patient in front of them, with the implicit and explicit expectations of the practice. This further reveals the essential dichotomy of routine practice – decisions conveyed as protocolised, without human involvement, can be reflective of complex layers of mainstream discourse, institutional bias, and interpersonal discrimination [1, 34–37, 39, 54, 63, 70]. 

There will always be patients who are miscategorised: people who are not entitled to free NHS care who are granted it, and patients who are eligible for care who are denied it. It is worth asking which of these miscategorisations we are more concerned in preventing. This is depicted in Table 3. UK Government policy, mirroring anti-migrant sentiment in many other nations, fixates on preventing Type 1 Errors (ineligible patients who are granted care), without considering that the efforts to do so may well increase the number of Type 2 errors (eligible patients who are denied care), and pursuing this approach is likely to have greater health and financial costs [8, 16, 62, 89, 90]. 

**Table 3:** Possible decision errors when registering a patient for free NHS care

	Deemed eligible for care	Deemed ineligible for care
Eligible for care	Correct	Type 2 Error “False Negative”
Ineligible for care	Type 1 Error “False Positive”	Correct

With the increasing pressure to charge migrants, ambiguity may enable (or preclude) access [11, 17, 60, 66, 83, 90]. It is therefore important to understand how structural racism and practice dynamics may influence how personal discretion is applied. Recent qualitative work with asylum seekers in England concluded that “the racialisation of healthcare is fueled by a politically racist policy agenda, accompanied by complex and often opaque healthcare entitlements for asylum seekers, which together legitimate misinformed and, at times, prejudicial attitudes within the NHS.” [91] This fits with a wider literature on how institutional racism is enacted in everyday healthcare encounters [92, 93]. 

It is important to highlight that reception work is classed as non-professional, and receptionists are among the lowest paid members of staff in the NHS, earning close to the minimum wage [26, 32]. Their role is undervalued – considered peripheral and subordinate to the work of a doctor – due to its historic feminisation, despite ample evidence of the complexity of the work it entails [94]. Reception work is also increasingly complex as more tasks are delegated to them [95]. The nature of these tasks is more akin to clinical decision making, rather than administrative work [96, 97]. They frequently experience abuse and complaints from patients and families. This can lead to staff becoming hardened over time and can negatively impact receptionist-patient interactions generally [27, 32, 34]. 

Research from the patient perspective shows they feel receptionists overstep the boundaries of their role and patients must often negotiate to receive care [26, 32, 34]. The reception area is seen as a space of constraint, not care or connection [86]. When protocols are written with only mainstream patients in mind, marginal patients (in this case, migrants) pose a threat to order and efficiency, as their vulnerabilities and needs introduce complexity. Receptionists may rationally prioritise keeping order, over striving for social justice, in a system that rewards the former and punishes the latter.

However, there is consistent research to suggest receptionists do possess increased flexibility for patients they consider vulnerable [26, 41, 54, 59]. Their interactions with different patient groups can therefore reflect community prejudices [30]. This is shown in ethnographic research in a clinic in the US colony of Guam, with indigenous Guamanian residents (the Chomorro) working as receptionists, but with predominantly immigrant Chukkese patient populations [30]. The reception staff created narratives around deservingness to care, that were reflective of wider community tensions. Other research from Canada and New Zealand mentions the gap between entitlement and access, and the role of the health care administrators in this [98, 99]. 

In short, our results link the decisions of frontline workers to structural factors. But whether, and where, to pin the blame is not the point. Lipsky himself asks us to balance structural and individual critique:

“This is not to say that one can easily strike out for one explanation of responsibility over another. Structural explanations of clients’ circumstances are important in order to direct attention to changing the political, economic, and social structures that circumscribe and dictate the possibilities of action. […] [But] in important respects [workers] to some degree must be responsible for themselves … [else] there can be no [worker] growth within the current structure of arrangements and no [worker] contributions to changing those arrangements, individually or collectively.” ([21], pg 154)

We recommend further ethnographic and sociological work to explore these relations in more detail.

**Fig. 3:**
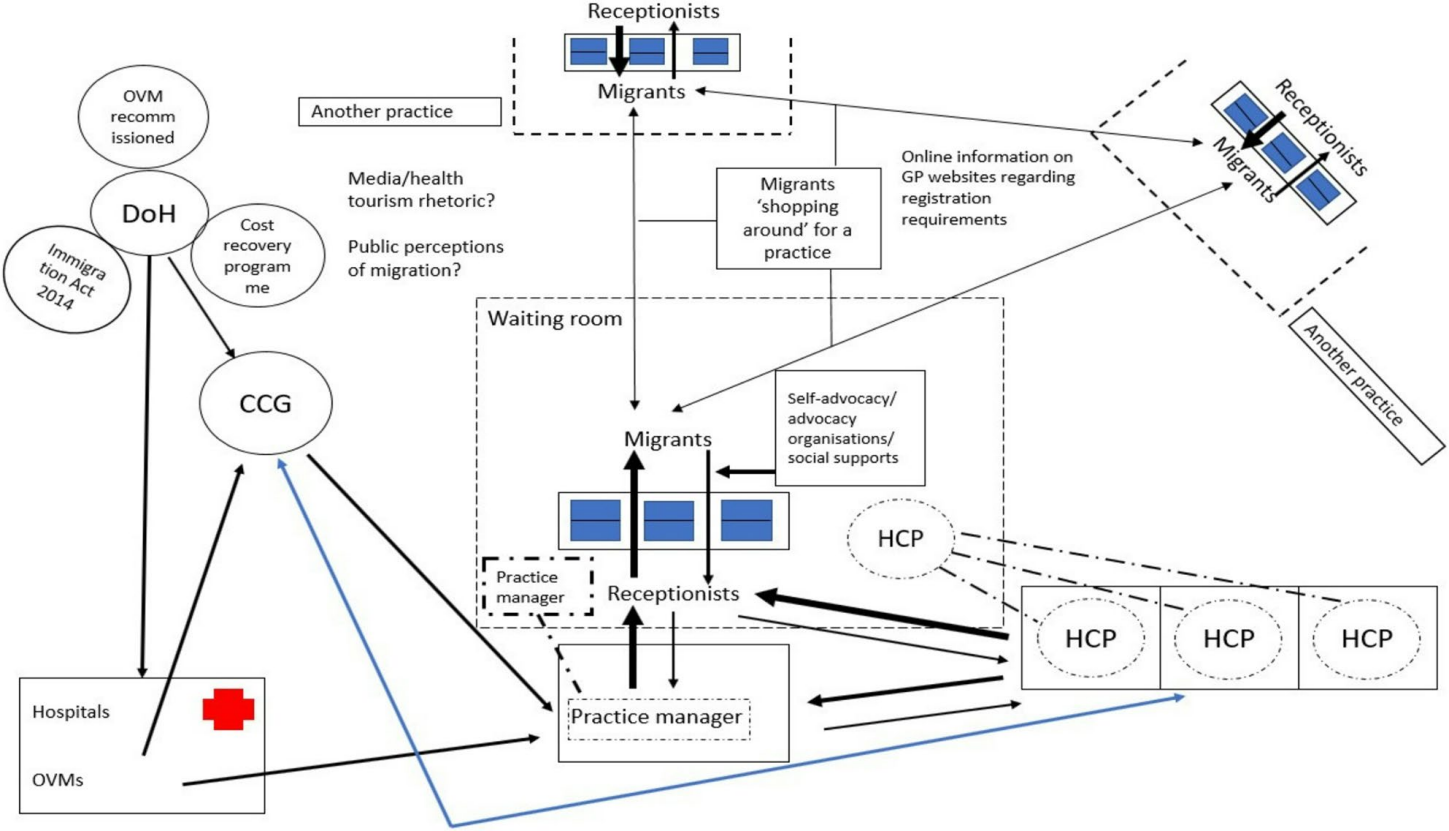
Logic model visually depicting the relevant social relations within the general practice reception space. This diagram summarises our results regarding the interactions within the practice, especially those between the receptionist and migrants. It attempts to highlight that receptionists’ decisions regarding migrants’ access are influenced by more than what takes place in the space of that interaction, but also that they operate within a relatively autonomous space separated from other practice staff. Receptionists’ interactions with migrants will reflect personal views, whilst also shaped by wider media representations of migrants and broader political discourse. Inconsistent practice requirements communicated by receptionists in an area may lead to migrants ‘shopping around’ for practices, hoping the will eventually find one that will register them [11, 12, 27–30, 35–37, 39, 54–56, 59, 61–64, 67–71, 74, 76–78, 98, 99, 102]. Solid lines demarcate spaces with restricted accessibility. Dashed lines indicate spaces, which can be accessed by the public. Where dashed lines surround individuals, it suggests they can transiently enter into accessible spaces. In this case HCPs and practice managers are generally in closed off spaces, but can choose to enter into the reception space if they wish to do so. Arrows indicate the level of influence between stakeholders. Thicker arrows indicate a higher level of power within that interaction. CCG = Clinical Commissioning Group; DoH = Department of Health and Social Care; HCP = Health Care professional; OVM = Overseas Visitor Manager (responsible for identifying and verifying the eligibility of patients who are not ordinarily resident in the UK for free NHS treatment

This review echoes the voices of migrants’ stories regarding their attempts to access GP and other NHS services [35, 63, 71, 73]. We feel our results are timely and relevant since restrictive entitlements and immigration policies are being implemented across Europe as right−wing populist sentiments gain traction [5, 11, 16, 44, 100, 101]. Receptionists and administrative staff should be considered critically in their role in this, as they will be expected to enforce both local and national policies regarding changing entitlements [13, 14, 60]. A logic model, depicting the reception space and how it relates to migrants trying to access care, is shown in Fig. 3.

## Strengths and limitations

This scoping review adopted a transparent method and followed the internationally recognised Joanna Briggs Institute method. An extensive literature search was conducted, which included seven scholarly databases as well as searches of websites and newspapers to identify grey literature. This allowed us to compile sociologically-relevant articles relating to our research aim. Our review is also theoretically well-informed, drawing on the relevant SLB theory, which has been under-applied to healthcare. Our authors included practising GPs, whose experience working alongside receptionists results in an equally sympathetic and critical approach.

There are several limitations to this study. First, the data analysis was done by the lead author alone. While we believe this helps with the coherence and meaning of our results, it opens our research up to certain biases, notably researcher bias and interpretation bias.

Second, the research includes articles preceding 2020, in order to capture normal practices and not pandemic-related upheavals [103]. Whilst we feel this makes the results still applicable to the majority of primary care settings in industrialised nations, this date range necessarily means it does not consider the increased digitalisation of primary care and how this has changed the receptionist’s role. Since 2020, ‘Total Triage’ has been introduced in many parts of the UK [26, 40]. This has changed the receptionist’s role. It limits the capacity for personal discretion, instead relying on algorithms. However, besides potentially worsening digital exclusion, it is precisely receptionists’ ability to apply personal discretion that allows them to positively advocate for vulnerable patients [26, 32, 41, 59, 73, 104]. 

Finally, our analysis did not consider the local sociodemographic contexts of the different GP practices mentioned; since socio-economic deprivation strongly determines workplace resources, staff burnout, and patient complexity, this might leave out a crucial piece of the story.

### Implications for practice

Primary care receptionists are the gateway to all healthcare, and whilst their work is taken for granted, the importance of their social role has gone under-explored. If gatekeepers are preferentially refusing access to healthcare to people based on their identity, then we must consider any means to make this process more objective. Our results lead us to propose five key recommendations for practice.

First, it should be centrally-mandated that all primary care clinics meet minimum standards with regards to being migrant-friendly [54, 83, 104]. Currently, a tiny fraction of GPs are official “safe surgeries”, which requires periodic, minimal audits and education interventions designed by the advocacy group ‘Doctors of the World.’ [83] Our results suggest that by making such training and checks mandatory for all existing and incoming reception staff, implicit bias in how migrants are treated will be reduced [83]. The existing Care Quality Commission inspections provide a place where such checks could be made. As it stands, barely a quarter of GP receptionists receive any training in triage, let al.one on how biases might shape triage [31]. This is important because Lipsky highlights how stereotyping of clients occurs as SLBs seek to simplify complex problems [18]. He notes that this can be inaccurate and prejudicial, reflecting prevailing biases in wider society. To quote Lipsky at length:

workers’ attitudes and resulting behavior may be challenged and helped to change if: incentives and sanctions within the structure of the job encourage change; the structure of the job is altered to reduce workers’ needs for psychological coping mechanisms; it can be shown that workers can cope successfully with job stresses without depending upon undesirable simplifications; efforts are made to make simplifications conform to actual job requirements rather than to unrelated biases ([21], pg 142)

But how can we stamp out *explicit* bias? Our second recommendation is that formal practice inspection is balanced by more informal, impromptu visits by “fake patients” who are actually paid practice inspectors. Such “secret shopper” inspections, if done without warning, would enable healthcare access to be gauged in several areas, not just with regards to migrants. This has been done in other settings [26, 41, 68, 105, 106]. The aim would be that individuals displaying discriminatory behaviour, would be met with remediation, and steps would be taken to address workplace ethos, as well as wider efforts to make the broader mainstream discourse more migrant friendly.

Thirdly, hiring staff reflective of the whole community, not only in terms of ethnicity, but also migration status and socio-economic background, would help to reduce bias, as would involving experts-by-experience to train staff [35, 38, 41, 63, 107, 108]. Other structural barriers should also be addressed, such as the lack of available translated materials and interpreter services for receptionists [109]. Services being moved online can partially address language barriers, as people can more easily employ online translation services. However, caution should be taken with regard to digital exclusion and accessibility of internet and computer services [40]. 

Our fourth recommendation is that all vulnerable migrants are offered, or even obliged to undertake, a registration process with a primary care clinic, facilitated by paid translators and community advocates. This exists in pockets in the UK (for example, ‘New Migrant Check’ appointments offered in one Birmingham practice), but there is currently no central support to make this provision sustainable and fairly-distributed [28]. Such “registration appointments” could be formalised. They could include basic education regarding the rights and responsibilities patients and the healthcare system have, in terms of keeping people healthy. This would have multiple benefits, as it would not only reduce the threshold of registration, but it would also help people to use the health system appropriately. This is particularly important for newly-arrived migrants, who may not be aware of how to navigate the complex health system [41, 76, 79, 107]. Such ‘New Migrant Check’ appointments could ensure multimorbid health needs can be met; one digital tool, Health Catch-UP!, is currently being trialed in the UK [110].

Our final recommendation is that reception work is respected for the complex and demanding work that it is. This will first and foremost involve a much more rigorous and diverse package of training both before and after starting the role. This training should acknowledge the need for receptionists to be not just protocolised task-actioners but problem solvers who should feel empowered to use their initiative and the powers we have shown they possess [69, 74, 102, 111]. It will also require a raise to receptionist salaries, with salaries rising annually to reward experience. This should be paired with drives to recruit a bright and diverse reception workforce, to minimise the high staff turnover seen in reception roles and boost the ethos and morale of staff [26, 31, 32, 34]. 

## Conclusion

Receptionists act as street−level bureaucrats in their interactions with migrants and can exercise personal discretion within their realm. Discriminatory attitudes and practices (sometimes positive, but usually negative) are present on the front−line of UK general practice. As a result, migrants who have full entitlements to care, and who may have significant health needs, are denied treatment without even being registered to see a healthcare professional. This decision can be directly or indirectly based on the migrant’s perceived immigration status or race. Structural barriers can be used to disguise discretionary practice, including the lack of available translation services, and restrictive (and illegal) practice policies that require the presentation of proof of address and ID. These behaviours on the front−line will not affect all migrants equally. The dynamic within the practice, including the strength of relationships between GPs, practice managers and receptionists, and the ethos of the institution, will influence receptionists’ behaviour when confronted with decisions regarding migrants’ access to primary care.

Further research needs to be conducted into the reasoning of receptionists, in order to better understand the extent to which the decisions can be considered independent of the influence of other practice staff or reflective of a wider practice ethos. This is particularly important due to the suggestion to introduce charging into UK primary care. Administrative staff will be expected to play a pivotal role in the identification of chargeable migrants. It is likely that if charging is introduced, without critical acknowledgement of how incorrect and discriminatory practices take place on the front−line, access will deteriorate even further for migrants who remain entitled to primary care. The rhetoric of ‘health tourism’ and migrant abuse of the NHS must be challenged, given the lack of evidence it is a significant financial burden on the NHS and the more likely xenophobic basis for its pursuit politically. It is crucial that the recommendations we make are considered in governmental policy, if primary care is to fulfil its clinical and ethical responsibility: to provide health care that is universal and equitable to all.

## Supplementary Information

Supplementary Material 1: S1 File. Full search strategy for three example databases. Online databases covering: scholarly and grey literature (Global Health); scholarly journal archives (British Journal of General Practice), and news databases (BBC News).

## Data Availability

The datasets used and/or analysed during the current study are available from the corresponding author on reasonable request.
