# Supplementary material for: Primary care receptionists influence migrant access to healthcare by acting as street-level bureaucrats: a scoping review
Source: BMC Prim Care. 2026 Feb 28;27:119. doi: 10.1186/s12875-026-03201-z (PMC13064384; doi:10.1186/s12875-026-03201-z)
Supplement: Supplementary file 1 — Supplementary Material 1: S1 File. Full search strategy for three example databases. Online databases covering: scholarly and grey literature (Global Health); scholarly journal archives (British Journal of General Practice), and news databases (BBC News). [file 12875_2026_3201_MOESM1_ESM.docx]

**Appendix 1: Full Search Strategy for Global Health Database, British Journal of General Practice and BBC News**

**Global Health Database**

### Search number 1

(Receptionist* OR reception* OR clerk OR admin OR administrative OR non−clinical OR non clinical OR “street level bureaucra* OR “street−level bureaucra*”)

AND

(Migrant OR migrant (mh explode) OR immigrant OR asylum seeker* OR refugee (mh explode) OR refugee OR “failed asylum seeker*” OR “rejected asylum seeker*” OR “refused asylum seeker*” OR “undocumented migrant*” OR “irregular migrant*” OR “illegal immigrant*” OR “illegal migrant*” OR “migrant worker*” OR “economic migrant*” OR “recently−arrived migrant*” OR “recently arrived migrant*” OR “newly−arrived migrant*” OR “overseas visitor*” OR “European Union Economic Area migrant*” OR “EEA migrant*” OR “EU migrant*” OR “EEA national*” OR “central Europe” (mh explode) OR “eastern Europe*” (mh explode) OR “visible minority ethnic*” OR “visible ethnic minorit*” OR “black minority ethnic*” OR “black and ethnic minorit*” OR VME OR BAME OR BME )

AND

(Access OR access (mh explode) OR “right of access” (mh explode) OR eligibility OR eligible OR utilisation OR utilization OR registration OR affordability OR chargeability OR charge OR candidacy OR navigation OR permeability OR adjudication OR “operating conditions”)

AND

(“primary healthcare” OR “health services” (mh explode) OR “primary health care” (mh explode) OR “primary care” OR “health care” (mh explode) OR PHC OR “general practice” OR GP OR “GP practice*” OR “community healthcentre*” OR “community health centre*” OR “community practice” OR “family practice” OR “health centre” OR “walk−in clinic*” OR “walk in clinic*” OR “drop− in clinic*” OR “drop in clinic*” OR “walk−in centre*” OR “drop−in centre*” OR “drop in centre*”)

### Total results 3

**Search** **number** **2**

Migrant* OR migrant (mh explode) OR immigrant* OR asylum seeker* OR refugee (mh explode) OR “failed asylum seeker*” OR “rejected asylum seeker*” OR “refused asylum seeker*” OR “undocumented migrant*” OR “irregular migrant*” OR “illegal immigrant*” OR “illegal migrant*” OR “migrant worker*” OR “economic migrant*” OR “recently−arrived migrant*” OR “recently arrived migrant*” OR “newly−arrived migrant*” OR “overseas visitor*”

AND

Access OR “right of access” (mh explode)

AND

(“primary healthcare” OR “health services” (mh explode) OR “primary health care” (mh explode) OR “primary care” OR “health care” (mh explode) OR PHC OR “general practice” OR GP OR “GP practice*” OR “community healthcentre*” OR “community health centre*” OR “community practice” OR “family practice” OR “health centre” OR “walk−in clinic*” OR “walk in clinic*” OR “drop− in clinic*” OR “drop in clinic*” OR “walk−in centre*” OR “drop−in centre*” OR “drop in centre*”)

### Total results 20

**Search** **number** **3**

(Receptionist* OR reception* OR clerk OR admin OR administrative OR non−clinical OR non clinical

OR “street level bureaucra* OR “street−level bureaucra*”)

AND

(Registration (mh explode) OR registration) AND

(“primary healthcare” OR “health services” (mh explode) OR “primary health care” (mh explode) OR “primary care” OR “health care” (mh explode) OR PHC OR “general practice” OR GP OR “GP practice*” OR “community healthcentre*” OR “community health centre*” OR “community practice” OR “family practice” OR “health centre” OR “walk−in clinic*” OR “walk in clinic*” OR “drop− in clinic*” OR “drop in clinic*” OR “walk−in centre*” OR “drop−in centre*” OR “drop in centre*”)

### Total results 0

**Search** **number** **4**

“failed asylum seeker*”

### Total results 4

**Search** **number** **5**

(Migrant* OR migrant (mh explode) OR immigrant* OR asylum seeker* OR refugee (mh explode) OR “failed asylum seeker*” OR “rejected asylum seeker*” OR “refused asylum seeker*” OR “undocumented migrant*” OR “irregular migrant*” OR “illegal immigrant*” OR “illegal migrant*” OR “migrant worker*” OR “economic migrant*” OR “recently−arrived migrant*” OR “recently arrived migrant*” OR “newly−arrived migrant*” OR “overseas visitor*” )

AND

(Access OR access (mh explode) OR “right of access” (mh explode) OR eligibility OR eligible OR utilisation OR utilization OR registration OR affordability OR chargeability OR charge OR candidacy OR navigation OR permeability OR adjudication OR “operating conditions”)

AND

(“primary healthcare” OR “health services” (mh explode) OR “primary health care” (mh explode) OR “primary care” OR “health care” (mh explode) OR PHC OR “general practice” OR GP OR “GP practice*” OR “community healthcentre*” OR “community health centre*” OR “community practice” OR “family practice” OR “health centre” OR “walk−in clinic*” OR “walk in clinic*” OR “drop− in clinic*” OR “drop in clinic*” OR “walk−in centre*” OR “drop−in centre*” OR “drop in centre*”)

AND

(“United Kingdom” OR UK (mh explode) OR UK OR England OR Wales OR Scotland OR “Great Britain”

OR GB)

### Total results 71

**Search** **number** **6**

(“visible ADJ2 minority ADJ2 ethnic*” OR “visible minority ethnic*” OR BAME OR “black ADJ2 minority ADJ2 ethnic*” OR VME OR BME OR “black ADJ2 and ADJ2 ethnic ADJ2 minorit*”)

AND

(Access OR access (mh explode) OR “right of access” (mh explode) OR eligibility OR eligible OR utilisation OR utilization OR registration OR affordability OR chargeability OR charge OR candidacy OR navigation OR permeability OR adjudication OR “operating conditions”)

AND

(“primary healthcare” OR “health services” (mh explode) OR “primary health care” (mh explode) OR “primary care” OR “health care” (mh explode) OR PHC OR “general practice” OR GP OR “GP practice*” OR “community healthcentre*” OR “community health centre*” OR “community practice” OR “family practice” OR “health centre” OR “walk−in clinic*” OR “walk in clinic*” OR “drop− in clinic*” OR “drop in clinic*” OR “walk−in centre*” OR “drop−in centre*” OR “drop in centre*”)

AND

(“United Kingdom” OR UK (mh explode) OR UK OR England OR Wales OR Scotland OR “Great Britain”

OR GB)

### Total results 211

**Search** **number** **7**

“European Union Economic Area migrant*” OR “EEA migrant*” OR “EU migrant*” OR “EEA national*” OR “central Europe” (mh explode) OR “eastern Europe*” (mh explode)

AND

(Access OR access (mh explode) OR “right of access” (mh explode) OR eligibility OR eligible OR utilisation OR utilization OR registration OR affordability OR chargeability OR charge OR candidacy OR navigation OR permeability OR adjudication OR “operating conditions”)

AND

(“primary healthcare” OR “health services” (mh explode) OR “primary health care” (mh explode) OR “primary care” OR “health care” (mh explode) OR PHC OR “general practice” OR GP OR “GP practice*” OR “community healthcentre*” OR “community health centre*” OR “community practice” OR “family practice” OR “health centre” OR “walk−in clinic*” OR “walk in clinic*” OR “drop− in clinic*” OR “drop in clinic*” OR “walk−in centre*” OR “drop−in centre*” OR “drop in centre*”)

AND

(“United Kingdom” OR UK (mh explode) OR UK OR England OR Wales OR Scotland OR “Great Britain”

OR GB)

### Total results 72

**BJGP** **Journal**

**Search** **number** **1** migrant NHS receptionist **Total** **results** **28**

### Search number 2

migrant access GP primary care receptionist

### Total results 28

**Search** **number** **4**

EU migrant access GP receptionist

### Total results 28

**Search** **number** **5**

European Union access GP receptionist

### Total results 50

**Search** **number** **6**

bame access GP receptionist

**Total** **results** **0**

**Search** **number** **7**

Black and minority ethnic access GP receptionist

**Total** **results** **641** However, when sorted by Best Match, apart from the first result, the results on the first 3 pages were irrelevant.

**BBC** **News** **Website**

**Search** **number** **1** migrant receptionist NHS **Total** **results** **0**

**Search** **number** **2** refugee receptionist NHS **Total** **results** **0**

**Search** **number** **3** refugee receptionist GP **Total** **results** **0**

**Search** **number** **4** refugee receptionist **Total** **results** **0**

**Search** **number** **5** refugee GP access **Total** **results** **0**

### Search number 6

Asylum seeker primary care

### Total results 3

**Search** **number** **7** refugee primary care **Total** **results** **3**
